# Dysconnectivity of the Agency Network in Schizophrenia: A Functional Magnetic Resonance Imaging Study

**DOI:** 10.3389/fpsyt.2019.00171

**Published:** 2019-04-03

**Authors:** Akihiro Koreki, Takaki Maeda, Tsukasa Okimura, Yuri Terasawa, Toshiaki Kikuchi, Satoshi Umeda, Shiro Nishikata, Tatsuhiko Yagihashi, Mari Kasahara, Chiyoko Nagai, Yasushi Moriyama, Ryosuke Den, Tamotsu Watanabe, Hirotsugu Kikumoto, Motoichiro Kato, Masaru Mimura

**Affiliations:** ^1^Department of Neuropsychiatry, School of Medicine, Keio University, Tokyo, Japan; ^2^Center for Psychiatry and Behavioral Science, Komagino Hospital, Tokyo, Japan; ^3^Department of Neuropsychiatry, National Hospital Organization Shimofusa Psychiatric Medical Center, Chiba, Japan; ^4^Department of Psychology, Keio University, Tokyo, Japan; ^5^Department of Psychiatry, Komagino Hospital, Tokyo, Japan; ^6^Department of Speech-Language Pathology and Audiology, Teikyo Heisei University, Tokyo, Japan

**Keywords:** schizophrenia, sense of agency, self-disturbance, functional connectivity, imaging, caudate, inferior parietal lobule, insula

## Abstract

**Background:** Self-disturbances in schizophrenia have recently been explained by an abnormality in the sense of agency (SoA). The cerebral structures of SoA in healthy people are considered to mainly include the insula and inferior parietal lobule. In contrast, the functional lesion of aberrant SoA in schizophrenia is not yet fully understood. Considering the recent explanation of establishing SoA from the standpoint of associative learning, the “agency network” may include not only the insula and inferior parietal lobule but also the striatum. We hypothesized that aberrant SoA in schizophrenia is based on a deficit in the “agency network.”

**Methods:** Functional magnetic resonance imaging data were acquired while patients with schizophrenia (*n* = 15) and matched controls (*n* = 15) performed our adaptation method of agency attribution task on a trial-by-trial basis to assess participants' explicit experience of the temporal causal relationship between an action and an external event with temporal biases. Analysis of functional connectivity was done using the right supramarginal gyrus and the right middle frontal gyrus as seed regions.

**Results:** In healthy controls, analyses revealed increased activation of the right inferior parietal lobule (mainly the supramarginal gyrus), right insula, and right middle frontal gyrus as an activation of the agency condition. We defined activated Brodmann areas shown in the agency condition of healthy controls as the seed region for connectivity analysis. The connectivity analysis revealed lower connectivity between the head of the left caudate nucleus and right supramarginal gyrus in the patients compared to healthy controls.

**Conclusions:** This dysconnectivity of the agency network in schizophrenia may lead to self-disturbance through deficits in associative learning of SoA. These findings may explain why pathological function of the striatum in schizophrenia leads to self-disturbance.

## Introduction

Alteration of self-consciousness has long been noted as a core feature of schizophrenia; the symptoms are generally referred to as “self-disturbances” (1). Self-disturbances in schizophrenia have recently been explained by abnormalities in the sense of agency (SoA), which is the attribution of oneself as the cause of one's own actions and their effects (2–7). SoA is selected as a subconstruct of the “social processes” domain in the Research Domain Criteria (8).

The cerebral structures of SoA in healthy people are considered to mainly include the insula and inferior parietal lobule (IPL) (8–11). In addition, some studies have shown that SoA is associated with the right side of these regions (12, 13). Our previous research also showed that SoA is associated with some regions including the insula and IPL (14). In contrast, the functional lesion of aberrant SoA in schizophrenia is not yet fully understood. Some studies have demonstrated functional abnormalities in the insula and IPL, which are associated with impaired self-other distinction (15–18). In contrast, some studies that evaluated SoA in schizophrenia using a SoA task showed abnormalities in frontal regions (19, 20). Therefore, the functional lesion of aberrant SoA in schizophrenia remains controversial. One reason for this controversy is that few brain imaging studies have directly targeted SoA in people with the illness.

In terms of computational accounts, the prevalent theory regarding the mechanism of SoA is the forward model (21, 22). In this model, whether the prediction of action matches the actual sensory consequence is important to develop SoA. An action consequence is predicted before the action execution. Following the action, the sensory data relating to the actual action consequences is compared with that of the prediction. If a match is present, the consequence is recognized as self-generated and SoA arises. If a mismatch is present, the consequence is recognized as externally generated and SoA is lost.

Recently, attention has been directed to an explanation for SoA from the standpoint of associative learning (23). Individuals develop SoA through the adaptiveness of perceiving controllability over their environment. Two types of associative learning have been described; Pavlovian learning (classical conditioning) and instrumental learning (operant conditioning). Instrumental learning consists of goal-directed learning and habit learning (24). Considering the adaptiveness of perceiving controllability in SoA, SoA is deeply associated with instrumental learning, especially goal-directed learning including reinforcement learning (23, 25). In the learning process, perceiving controllability is desirable and could be inherently rewarding (23).

The striatum plays a pivotal role in associative learning (24). A functional division of the striatum has recently been clarified. The striatum consists of the ventral striatum and dorsal striatum. The ventral striatum (mainly the nucleus accumbens) is involved in Pavlovian learning and is activated when a reward is unpredictable (24, 26). In contrast, the dorsal striatum is involved in instrumental learning. The dorsal striatum consists of the associative striatum [all of the caudate nucleus (CN) and the anterior part of the putamen] and the sensorimotor striatum (all of the putamen except for its most anterior portion). The associative striatum is involved in goal-directed learning and is activated when the reward is predictable, i.e., when a perception of contingency exists between action and outcome (26–29). The sensorimotor striatum is involved in habit learning and plays a role in the transition from goal-directed behavior to habitual behavior (25–29). Therefore, the associative striatum may play an especially important role in the emergence of SoA. In fact, Graybiel indicated that the striatum could provide a mechanism for the development of self-agency, that is, the striatum may function in the monitoring of intentional actions and their consequences, and, as a result, participate in the development of motor and cognitive patterns that differentiate self from others (30, 31).

Regarding schizophrenia, the striatum has been reported to be one of the most critical regions in the pathophysiology of symptoms (32, 33). Psychopharmacological studies have advocated for the dopamine hypothesis in which excessive dopamine in the striatum leads to positive symptoms including self-disturbance. This hypothesis is robustly supported by recent positron emission tomography (PET) studies showing increased availability of D2 receptors in schizophrenia (34). A recent PET study demonstrated a greater increase in D2 receptors in the dorsal striatum than in the ventral striatum, indicating the importance of the dorsal striatum in schizophrenia (34). However, how abnormalities in the dorsal striatum affect aberrant self-disturbance is unclear.

Although previous studies have mainly focused on the neural localization of the pathological mechanism in schizophrenia, the neural network connecting these regions should also be investigated (35, 36). Therefore, we hypothesized that aberrant SoA in schizophrenia is based on deficits in the “agency network,” including the insula, IPL, and striatum. To investigate network deficits in schizophrenia, evaluation of functional connectivity using functional magnetic resonance imaging (fMRI) is essential. However, few papers have evaluated abnormal functional connectivity linked to aberrant SoA in schizophrenia. Backasch et al. showed low functional connectivity between the inferior frontal gyrus and some regions (the insula, putamen, and medial orbitofrontal cortex) in schizophrenia by using their own agency task with unintentional movement and delayed visual feedback (20). Spaniel et al. demonstrated an impaired default mode and central executive networks dynamic switching in schizophrenia by using their own agency task with incongruent visual feedback (37). Although their task designs were useful for evaluating one neural network, they did not target the learning process in agency adaptation. Therefore, since individuals develop SoA through associative learning, a task which evaluates the adaptation process of SoA is needed (23). We have investigated SoA in schizophrenia using our original agency attribution task to assess participants' explicit experience of the temporal causal relationship between an action and an external event with random temporal biases (5, 6). Furthermore, we designed the trial-by-trial method of the task, which is designed to force participants to learn temporal causal relationships between an action and a subsequent event as associative learning (7, 14). In this trial-by-trial method, the temporal bias in each subsequent trial is set based on the participant's response in the preceding trial (7, 14).

Therefore, the evaluation of inappropriate agency adaptation is important to investigate an aberrant agency network in schizophrenia. In the present study, functional connectivity was compared between healthy controls and patients with schizophrenia by using our trial-by-trial agency task as an exploratory study.

## Methods

### Participants

The participants were 15 patients (age 41.5 ± 13.9 years, six females, 11 outpatients; all right-handed) with schizophrenia classified by the Diagnostic and Statistical Manual of Mental Disorder-IV-Text Revision criteria and 15 age- and gender-matched healthy controls. All patients had chronic schizophrenia and were taking antipsychotics at the time of scanning (11.9 ± 10.1 haloperidol equivalent mg). The Positive and Negative Syndrome Scale (38) and the Global Assessment of Functioning score (39) were used to assess their clinical status. Exclusion criteria were (1) major brain anomaly or organic brain disease; (2) current or past substance abuse, including alcohol; (3) mental retardation; and (4) previous episodes of a mood disorder. Healthy controls were confirmed to have no psychiatric nor neurological disorders, nor any first-degree relatives with neuropsychiatric disorders. Demographic characteristics of participants are shown in Table 1. The education level was different between the groups (*p* = 0.02). No patients dropped out or showed any change in their psychiatric state during or after the experiment. This study was approved by the Ethics Committee at Komagino Hospital. All participants gave written informed consent prior to participation.

**Table 1:** Demographic characteristics of participants.

	**Schizophrenia (*n* = 15)**	**Controls (*n* = 15)**	***p***
Age, years	41.5 (13.9)	44.5 (10.5)	0.50
Sex, male/female	9/6	6/9	0.46
Education, years	13.3 (4.1)	16.5 (2.9)	0.02
Outpatient/inpatient	11/4		
Duration of illness, years	18.3 (11.5)		
Neuroleptic dosage, HP^*^-mg	11.9 (10.1)		
GAF^**^	54.7 (13.4)		
PANSS^***^ (total score)	64.3 (21.0)		
Positive symptoms	16.7 (7.0)		
Negative symptoms	17.9 (5.4)		
General psychopathology	29.6 (10.4)		

*Values are presented as mean (standard deviation) unless otherwise noted. Healthy controls were matched with patients for age and sex*.

* HP, Haloperidol;

** GAF, Grobal Assessment Functioning;

*** *PANSS, Positive and Negative Syndrome Scale*.

### General Procedure

**Figure 1:**
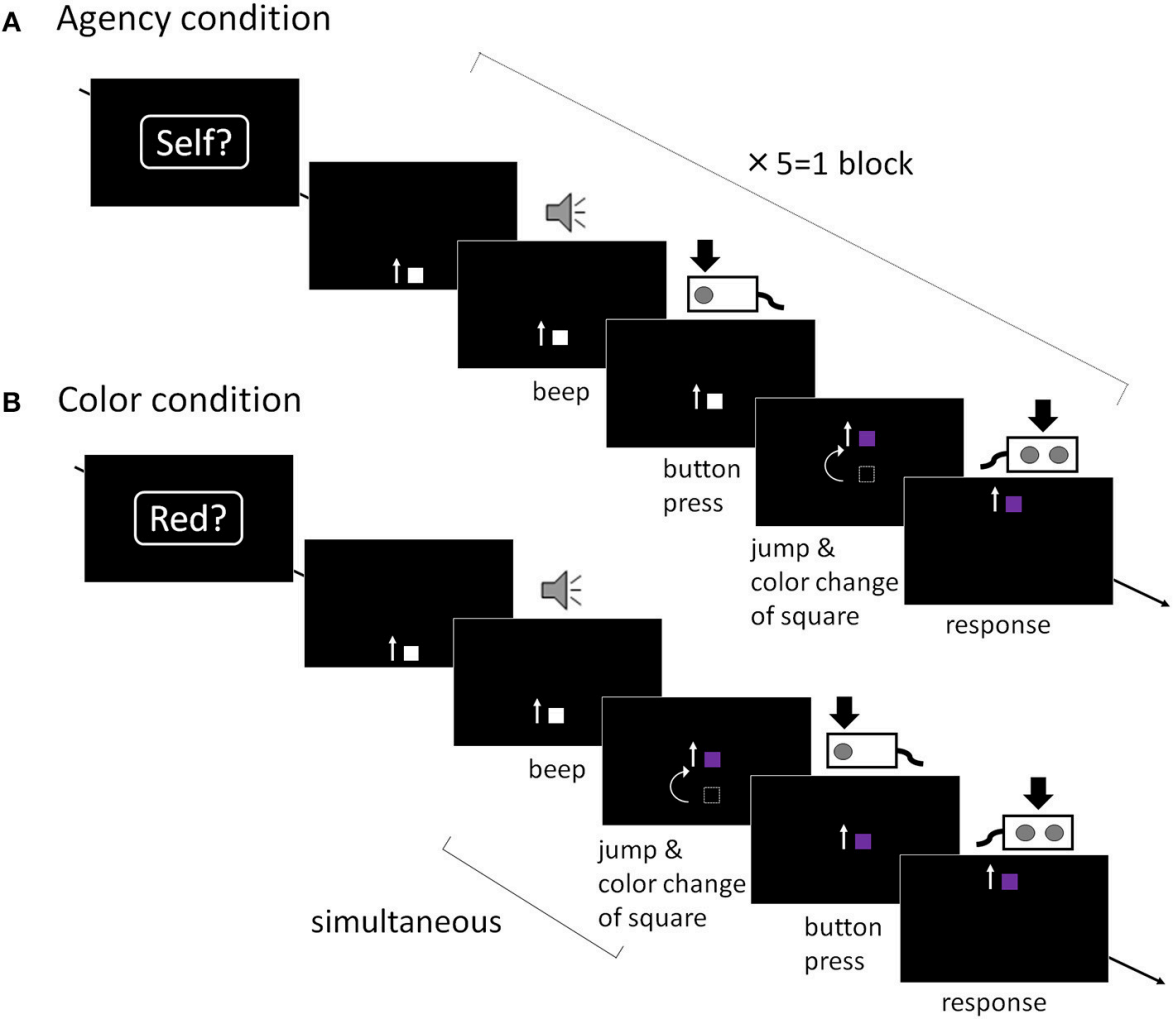
Trial sequence. **(A)** Agency condition: A moving square was presented and participants were required to press a button after they heard an auditory cue. After the button was pressed, the square jumped and the color changed with various temporal bias. Participants were then asked to report “whether I caused the square to jump or not (yes or no).” The temporal bias was initially set to 400 ms. This bias was set in each subsequent agency trial, depending on the participant's response in the preceding agency trial in accordance with a “trial-by-trial” method. When participants reported “Yes” (i.e., when they attributed the square's jumping to self-agency), the timing of the jumping in the next trial was extended by 50 ms. On the other hand, if they reported “No,” the delay in the succeeding trial was shortened by 50 ms. **(B)** Color condition: the square jumped and the color changed simultaneously and were linked to the auditory cue, not their button press. Participants were then required to report “whether the color of the square had changed to red or not (yes or no).” The color change in the succeeding color trial was set depending on the preceding color trial.

The present fMRI study was a block design, which consisted of agency and color conditions (as a control task) (Figure 1). Each block consisted of five trials with a duration of 7 s per trial of the same condition. Participants performed 20 blocks (each 50 trials in total), divided into two sessions. The order of the blocks was pseudo-randomized. Before experimental sessions, participants completed one block each both outside and inside the scanner for practice and to confirm that they performed the task successfully. The assignment of response keys in the task was fixed among sessions and participants. The experiment was controlled by E-prime software (Psychology Software Tools, Inc., Sharpsburg, PA, USA). The stimuli were projected on a 32-inch monitor (Nordic Neuro Lab, Bergen, Norway) with a mirror system mounted on the MRI head coil. The distance between participants' eyes and mirror was 215 mm. Participants held response boxes (HHSC-2 × 4-C, Current Designs, Inc., Philadelphia, PA, USA) in each hand and wore headphones (Serene Sound System, Resonance Technology Inc., Northridge, CA, USA).

### Stimulus Construction

#### Agency Condition

At the beginning of each agency block, a word (“Self?”) was presented as a prompt, indicating that this block would require a response regarding SoA. This word was presented for 1 s followed by a black screen for 500 ms. After this prompt, five agency trials were run in a row. In each agency trial, participants saw a 4 mm gray moving square, which appeared from the bottom of the black screen and moved straight upward at a uniform speed (6 cm/s). An auditory cue (1,000 Hz pure tone; 100 ms duration) was presented 2 s (±100 ms) after the square emerged, and participants were asked to press a button with their right index finger as quickly as possible when they perceived the auditory cue. The moving square on the screen jumped 8 cm upward and changed color, with various temporal bias after their button press. The square kept moving straight up and disappeared out of the display. Before the disappearance of the square, participants were required to report whether they felt that the square's jumping was caused by their own preceding action by using a response box in their left hand, pressing a button with the index finger to report “yes,” or a separate button with their middle finger to report “no.” This procedure from appearance to disappearance of the square was repeated five times. The temporal bias in each trial was set on a trial-by-trial basis in our experimental design. It initially set to 400 ms because a temporal bias of 400 ms in this paradigm was the boundary between self-other attribution in healthy participants (5–7), and was an appropriate starting point to promote brain activation and avoid biasing the participants' responses. In the second and the subsequent trial, this temporal bias was adjusted in each agency trial in the following manner. When participants reported “yes” in the previous agency trial (i.e., when they attributed the square's jumping to self-agency), the temporal bias of the jumping in the succeeding agency trial was extended by 50 ms compared with the previous agency trial. Conversely, if they reported “no,” the temporal bias in the succeeding agency trial was shortened by 50 ms. In contrast, color changes were independent of the participant's reports. The color also changed when the square jumped, but it was selected randomly from a range of 11 colors which was presented in the color condition to match the visual properties of the stimuli with the color condition described below.

#### Color Condition

Similarly to the agency condition, at the beginning of each color block, a word (“Red?”) was presented as a prompt, indicating that this block would require a response regarding the color of the square. After this prompt, five color trials were run in a row. The task sequence of the color trials was identical to that in the agency trials except for the timing of the change of the square. Participants were asked to press the button as quickly as possible when they perceived the auditory cue, but the square's jumps and color changes occurred simultaneously with the auditory cue, not their button press. This means that the color changes were causally linked to the auditory cue and occurred prior to their button press. Any aspect of SoA was eliminated in the color condition. Lastly, upon disappearance of the square, participants were asked to report whether the color of the square had changed to “red” by using a response box in their left hand in the same way as in the agency condition. This procedure from appearance to disappearance of the square was repeated five times. The color of the square was also set on a trial-by-trial basis. The set of color consists of a graded combination of red and blue in 11 steps. The reddest (color step: 1st) was coded as (200, 50, 150) and the bluest color (color step: 11th) was coded as (100, 50, 250) using decimal numbers in the 8-bit red-green-blue assignments (7, 14). The color adjustment in each subsequent color trial depended on the participant's response in the preceding color trial in the same manner as in the agency condition. When a participant reported “yes,” the color in the succeeding color trial was set one step toward blue. Conversely, when a participant reported “no,” the color was set one step toward red. The color was initially set to (160, 50, 190) (color step: 5th).

### Analysis of Behavioral Data

As mentioned above, the experimental setting of the temporal bias and color step was adjusted based on the participant's answer. Therefore, to check their adaptiveness for SoA and color, analysis of how the setting was adjusted is important. The adaptive courses of temporal bias and color step were analyzed using mixed effect models with a random intercept for participant. Group (healthy controls/patients with schizophrenia), trial, and group-by-trial interactions were modeled as fixed effects, with trial treated as a continuous covariate. As temporal bias was adjusted based on their previous trial, long temporal bias represents their excessive agency in schizophrenia. The longest temporal bias was defined as the longest value throughout the trials. Although they learnt and adjusted their agency throughout the trials, this can be one of the behavioral values representing their excessive agency. Therefore, in patients with schizophrenia, the longest temporal bias was also analyzed using Spearman correlation analysis to investigate the relationship to brain connectivity.

Pearson or Spearman correlation analysis was done between contrast estimates in the agency condition of patients and their clinical information.

In addition, reaction times (RTs) to beep sounds in all conditions were also analyzed using 2 way repeated measures ANOVA. Bonferroni correction was also done as a *post hoc* analysis. Statistical analyses were carried out using SPSS Version 23.0 (IBM Corp., Armonk, NY, USA).

### Functional MRI Data Acquisition and Analysis

Functional MRI scanning was performed with a 3T-MRI (Signa HDxt, GE Healthcare). Scanning consisted of two experimental functional runs, a T1-weighted structural scan (3-dimensional spoiled gradient echo sequence, slice thickness = 1.0 mm, TR = 6.8 ms, TE = 2.9 ms), and a T2-weighted structural scan (2-dimensional propeller scan, TR = 4,500 ms, TE = 98.3 ms). T2 images were used only to check that the participants had no obvious brain lesion. Each functional run consisted of whole-brain T2^⋆^-weighted single-shot gradient echo-planar images, collected in an oblique axial orientation [TR = 2,350 ms, TE = 30 ms, flip-angle = 90 degrees, voxel size of 3.5 mm × 3.5 mm × 2 mm, 44 slices (descending), slice gap = 1 mm].

To confirm the validity of our experimental setting, we analyzed activation of each condition. The acquired data set were preprocessed and analyzed using the SPM8 software package (Wellcome Trust Centre for Neuroimaging, London, UK). The initial three functional imaging data were discarded for the magnetization equilibrium. The structural imaging data was co-registered to the subject's mean echo-planar image. Functional imaging data were spatially corrected for head motion and temporally corrected for slice timing (using the middle slice acquired in time as a reference). Subsequently, the data were spatially normalized to the Montreal Neurological Institute template with a resample voxel size of 3.5 mm × 3.5 mm × 3.5 mm, and then spatially smoothed by an 8 mm full width at half maximum Gaussian. Furthermore, low-frequency drift in the signal was removed via high-pass temporal filtering with a cut-off of 128 s, and global changes were removed via proportional scaling.

We modeled each block (with a duration of 35 s) in the two conditions (agency/color conditions) as regressors with a canonical hemodynamic response function and its temporal derivative. General linear model was fitted to each contrast of the *t* statistic on a voxel-by-voxel basis to generate statistical parametric maps. To analyze the main effect, which means just significant activation in each condition compared to the baseline (fixation period), not a comparison of agency vs. color condition, a statistical threshold of *p* < 0.05, Family-Wise Error, with an extent threshold of two voxels, was used based on our previous study (14) because this analysis is about activation in each condition without comparison between conditions and has a great risk of false positive. As our experimental design was a block design that was selected to weigh analysis of functional connectivity and our analyses included many various components of the agency task, we selected the analysis of the main effect correlated to activation in each condition. Considering the difference between healthy controls and patients, the main effect was analyzed separately in each group. Contrasts between the condition and the groups were also analyzed.

To evaluate functional connectivity, the data were analyzed using a seed-driven approach with the CONN-fMRI Functional Connectivity toolbox (40). We selected the right insula and the IPL as seed regions for connectivity analysis based on our hypothesis and various previous studies (8–14). Moreover, RDoC defines these regions as a core of the neural basis of SoA because they are the most frequently and robustly activated regions in various agency associated tasks (8). Particularly, the right sides of these regions are more associated with SoA (12, 13). In addition, as analysis using various seed regions may yield false positive, we narrowed down the seeds to these two seed regions. The toolbox performed preprocessing (including head motion correction and segmentation), the first-level general linear model for correlation connectivity estimation, and the second-level random-effect analysis. We generated temporal connectivity maps for the agency condition by estimating the correlation coefficient between the seed signal and all other brain voxels. A cluster extent threshold of *p* < 0.05 was set for bidirectional explorations of connectivity as an exploratory analysis. To evaluate contrast estimates among the groups and the conditions, each contrast estimate was extracted into the SPSS and analyzed using a 2-way repeated measures analysis of variance. We did not set any factors as a covariate, for example, RTs is not simply reflected in difficulty of our agency tasks. Moreover, there is a risk of eliminating important factors which should be considered in this task. Bonferroni correction was also done as a *post hoc* analysis. In addition, Pearson or Spearman correlation analysis was done between contrast estimates in the agency condition of patients and their clinical information.

## Results

### Behavioral Results

**Figure 2:**
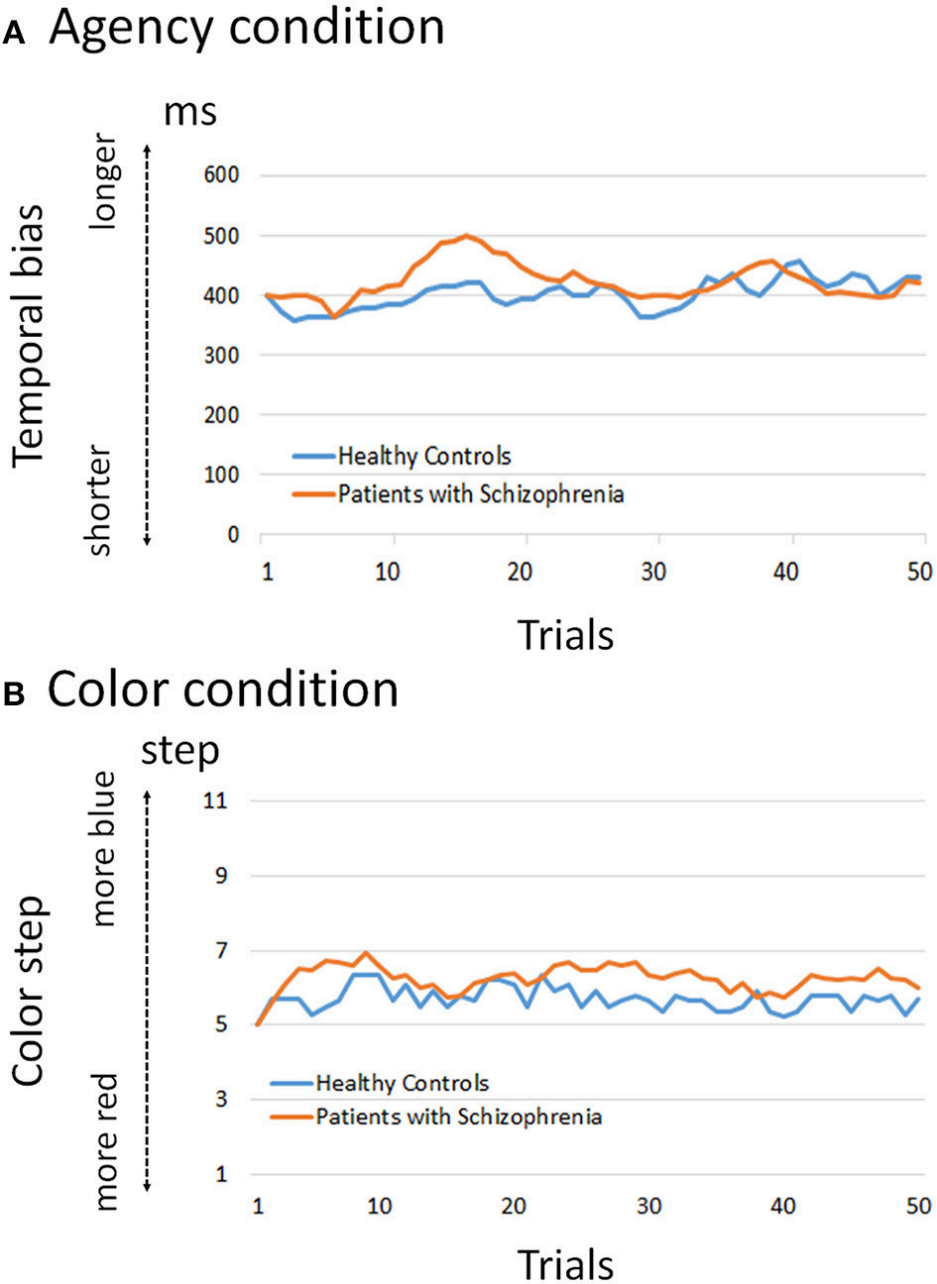
Adaptation course of temporal bias and color step. **(A)** The mixed effect model for the agency condition showed that the group-by-trial interaction was significant (*p* < 0.01), whereas group and trial were not significant (*p* = 0.315 and 0.684, respectively). This indicates that the values of temporal bias did not differ between healthy controls and patients, but the adaptive courses of agency with each trial were different. **(B)** The model for the color condition showed that group, trial, and group-by-trial interaction were not significant (*p* = 0.369, 0.711, and 0.347, respectively). This indicates that the adaptive courses of color showed no difference between healthy controls and patients.

Adaptive courses of temporal bias and color step are shown in Figure 2. The mixed effect model for the agency condition showed that the main effects of group and trial were not significant (*p* = 0.315 and 0.684, respectively) whereas the group-by-trial interaction was significant (*p* = 0.000). This indicates that the values of temporal bias did not differ between healthy controls and patients, but the adaptive courses of agency with each trial were different. The model for the color condition showed that the main effects of group and trial, and group-by-trial interaction, were not significant (*p* = 0.369, 0.711, and 0.347, respectively). This indicates that the adaptive courses of color showed no difference between healthy controls and patients.

RTs [mean (standard deviation)] during the agency condition in healthy controls and patients with schizophrenia were 358.9 (43.0) ms and 439.7 (93.8) ms, respectively. RTs during the color condition in healthy controls and patients with schizophrenia were 379.6 (110.9) ms and 436.5 (136.3) ms, respectively. The analysis for RTs in all conditions showed that the group was significant [*F*_(1, 28)_ = 4.291, *p* = 0.048], whereas condition and group-by-condition were not significant [*F*_(1, 28)_ = 0.272 and 0.507, *p* = 0.606 and 0.482, respectively]. *Post hoc* analysis revealed significant difference between the groups in agency condition (*p* = 0.042).

### Functional MRI Results

In the present study, the goal of analysis is to compare functional connectivity between healthy controls and patients with schizophrenia in the agency condition. Therefore, firstly, the main effect of the agency condition was analyzed. It was particularly evaluated in healthy controls to confirm the validity of our agency task and use a reference for selecting the seed regions for connectivity analysis. Secondly, we performed seed-region based connectivity analysis based on the seed region to investigate an aberrant agency network in schizophrenia.

### Main Effect of Each Condition

#### Agency Condition

**Figure 3:**
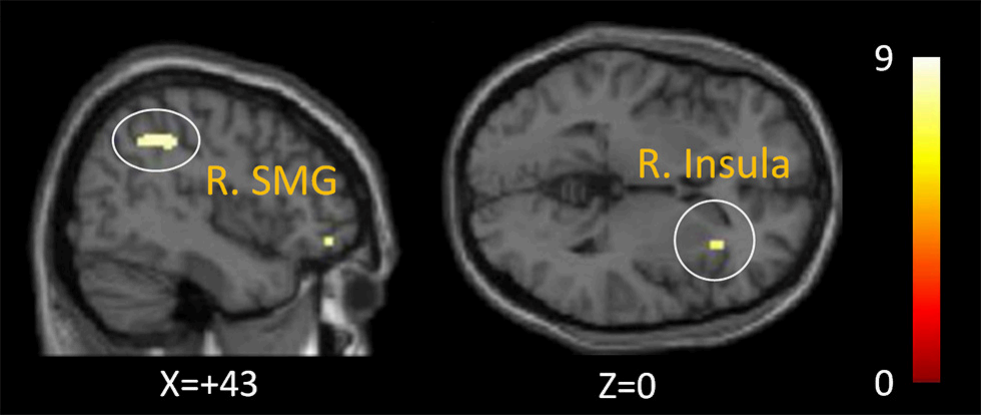
Activation of the agency condition in healthy controls. The right inferior parietal lobule (mainly supramarginal gyrus) and right insula was activated as a significant activation of the agency condition in healthy controls (*p* < 0.05, Family-Wise Error correction).

In healthy controls, analyses revealed increased activation of the right IPL [mainly supramarginal gyrus (SMG)], right insula, and right middle frontal gyrus as a main effect of the agency condition (*p* < 0.05, Family-Wise Error correction) (Figure 3). In patients with schizophrenia, the analyses revealed increased activation of the left supplementary motor area, left precentral gyrus, and left superior parietal lobule as a significant main effect of the agency condition (shown in Supplementary Table 1).

#### Color Condition

In healthy controls, no significant main effect of the color condition was found. In patients with schizophrenia, the left precentral gyrus, left postcentral gyrus, left supplementary motor area, right inferior frontal gyrus, left insula cortex, right middle frontal gyrus, right postcentral gyrus, and left superior parietal lobule were found as significant main effects (shown in Supplementary Table 1).

#### Contrast Between Groups and Between Conditions

There was no significant contrast region between agency and color conditions in each group nor between the groups in each condition.

### Analyses of Functional Connectivity

**Figure 4:**
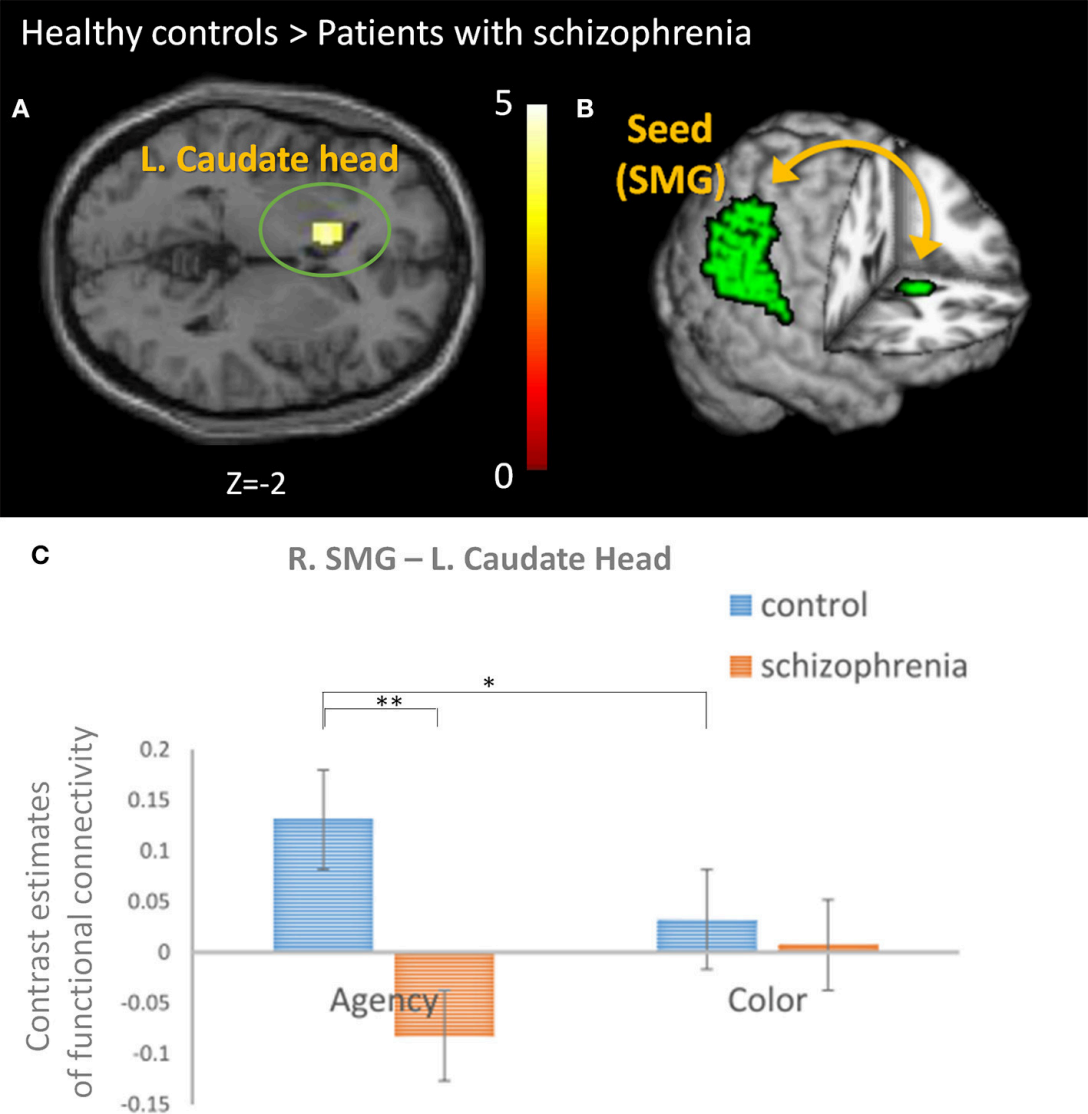
The comparison of the functional connectivity between groups. **(A)** Connectivity analysis revealed lower connectivity to the head of the left caudate nucleus (CN) from the right supramarginal gyrus (SMG) in patients with schizophrenia than healthy controls (*p* = 0.048, uncorrected, k_E_ = 144, a cluster extent threshold). **(B)** The analyses using a seed-driven approach revealed the importance of the functional connectivity between the right SMG (as a seed) and left CN for sense of agency (SoA) and the abnormality in schizophrenia. **(C)** The contrast estimates of the functional connectivity between the right SMG and left CN was compared. We found a significant difference in group and group-by-condition [*F*_(1, 28)_ = 8.552 and 10.648, *p* = 0.007 and 0.003, respectively]. *Post hoc* analysis revealed significant difference between the groups in agency condition (**p = 0.002) and between the conditions in healthy controls (*p = 0.042).

To evaluate the neural network associated with SoA in the agency condition, we firstly defined the activated Brodmann areas (right BA40 [SMG] and BA13 [insula]) as the seed regions for connectivity analysis based on our hypothesis. Compared to healthy controls, lower connectivity to the left CN head in the right SMG was observed in patients (*p* = 0.048, uncorrected, k_E_ = 144, a cluster extent threshold) (Figure 4). We found no significant difference in functional connectivity in the right insula.

We did not analyze specific functional connectivity to color condition because no significant region was observed in the color condition of healthy controls, but the same functional connectivity was analyzed as the agency condition for comparison between the conditions and the groups. The intergroup analysis for contrast estimates of this functional connectivity in both the conditions revealed that the group and group-by-condition were significant [*F*_(1, 28)_ = 8.552 and 10.648, *p* = 0.007 and 0.003, respectively], whereas condition was not significant [*F*_(1, 28)_ = 0.025, *p* = 0.875] (Figure 4). *Post hoc* analysis revealed significant difference between the groups in agency condition (*p* = 0.002) and between the conditions in healthy controls (*p* = 0.042).

### Correlation to Clinical Information and Behavioral Data

Contrast estimates in the agency condition of patients were significantly correlated with PANSS-G (*r* = −0.588, *p* = 0.021), and had trend-level correlation with PANSS-P (*r* = −0.481, *p* = 0.070) in the Pearson correlation coefficient analysis, while duration of illness (*r* = −0.089, *p* = 0.752), GAF (*r* = 0.369, *p* = 0.176), or PANSS-N (*r* = −0.380, *p* = 0.162) were not significant in Pearson correlation coefficient analysis. Contrast estimates also had trend-level correlation with neuroleptic dosage (*r* = −0.455, *p* = 0.088) as per the Spearman correlation analysis. There is no significant correlation with the longest temporal bias.

## Discussion

This study investigated the abnormal agency network in schizophrenia. To the best of our knowledge, this is the first report showing a deficit in functional connectivity between the right SMG and left CN in patients with schizophrenia associated with SoA. Considering the comparison of the connectivity between agency and color conditions, this connectivity is specific to SoA. SMG is a core region in the agency network, and CN is a core pathological region in schizophrenia as shown in psychopharmacological studies (8, 33). In fact, recent studies have shown an association between the CN and perception of control, perception of contingency between actions and outcomes, and self-serving bias, which are relevant to SoA (23, 26, 41). Therefore, disconnection in the agency network involving CN may lead to aberrant SoA and our finding may provide an integrated explanation for why a pathological function of the striatum in schizophrenia leads to positive symptoms including self-disturbance. Thus, our findings about correlation between contrast estimates and PANSS-G and P may partially support this link.

First, an activated right insula and right IPL in healthy controls during the agency condition would support the validity of our agency task because these regions have been reported as core SoA regions (8, 9, 14).

Furthermore, we found significantly weaker functional connectivity between the right SMG and left CN in schizophrenia compared to healthy controls. In terms of neural basis of learning process for SoA, the CN plays a pivotal role in goal-directed learning including reinforcement learning, and is activated when a perception of contingency exists between an action and its outcome (23, 26). Through the process of goal-directed learning, individuals achieve adaptation of SoA that is appropriate for their environment (23). Perceiving controllability is desirable and could be inherently rewarding (23). Therefore, our result suggests that patients with schizophrenia have difficulty achieving adaptation of SoA under uncertain conditions using the trial-by-trial method. In fact, our behavioral data showed a different adaptive course during the agency condition, suggesting an aberrant learning process in schizophrenia. Particularly, in the early phase of our graph, patients with schizophrenia tend to exhibit a SoA in consecutive trial-by-trial trials which suggests that they exhibited SoA even in trials with prolonged temporal delay). This tendency is in accordance with our previous study (7). Moore et al. also showed that people with schizotypy perform poor associative learning in an intentional-binding task, indicating the possibility of disrupted instrumental learning in schizophrenia (25).

The computational account regarding the mechanism of SoA is the forward model, which was originally proposed from a theoretical concept in motor learning (22, 42). In motor learning, to adapt internal models to an environment, the difference between predicted and actual sensory feedback can be used as an error signal to update a predictive model (43). Similarly, prediction in the forward model may be updated by actual sensory feedback to adopt a SoA that is appropriate for the environment. Actually, mathematical analysis of the data obtained from healthy controls in our previous SoA study showed that the forward model is updated through the Bayesian learning algorithm for the predictive distribution (44). Considering the updating mechanism, if updating of the forward model for SoA is impaired, aberrant SoA may result. In fact, several studies have shown evidence for impaired prediction signals in the forward model in patients with schizophrenia (7, 45, 46).

The abnormalities of the CN in schizophrenia have been shown in many studies (34, 47–49). A recent PET study demonstrated the importance of the CN in schizophrenia (34). Kreczmanski et al. showed a reduced total number of neurons in the CN using analysis of post-mortem schizophrenia brain (47). Using voxel-based morphometry, Tao et al. showed that patients with delusions of reference have reduced gray matter density in the CN compared with patients without delusions and healthy controls (48). Mueller et al. demonstrated abnormalities in the hemispheric specialization of the CN in schizophrenia from the point of view of disrupted neurodevelopment using resting state fMRI (49). Moreover, Huntington disease is a representative illness with pathology in the CN. Intriguingly, patients with early-stage Huntington disease claim that their choreic movements are voluntary (50). Kranick and Hallet state that this phenomenon is similar to excessive SoA in patients with schizophrenia who paradoxically over-attribute some events to the consequence of their own actions (50). Clinically, patients with Huntington disease often show schizophrenia-like symptoms (51). In addition, the pathophysiology of Huntington disease involves excess dopamine in the striatum, and antipsychotics are often used to treat chorea (52). These similarities may support the notion that pathology in the CN plays an important role in aberrant SoA in schizophrenia. In terms of pathophysiological perspective of schizophrenia, our results in this study could explain the pathological roles of CN for psychotic symptoms including self-disturbances.

In the present study, interhemispheric connectivity should be discussed. SoA is associated with the insula and IPL, especially on the right side (8, 12, 13). The right IPL is not limited to SoA and is associated with various self-relevant tasks, including cognition of one's own face and distinction between self and other (53, 54). In contrast, the function of the CN may be left dominant (49, 55). Using resting-state fMRI, Mueller et al. showed that the left CN is more specialized than the right CN in healthy controls (49). Interestingly, the lateralization was diminished in schizophrenia (49). A meta-analysis investigating the laterality of subcortical regional volumes demonstrated leftward laterality for the CN in both healthy controls and patients with schizophrenia (55). Therefore, the laterality shown in the present study is compatible with the laterality of the IPL and CN. Furthermore, deficits in interhemispheric connectivity have been reported in schizophrenia. Callosal and other cortico-cortical white matter tract impairments may be central to the illness (56).

The IPL and CN are also associated with time cognition (57–59). A recent study demonstrated that the front-striato-parietal network is associated with ms-s time perception (60). However, Kranjec et al. showed that causality is more significantly associated with the left CN than time and space (61). Causality is an important factor for SoA and is relevant to a perception of contingency that exists between action and outcome in goal-directed learning. Therefore, in the present study, we consider that the connectivity between these regions was associated with SoA, not just time cognition.

We believe that reduced functional connectivity in patients is not due to the difference of the local activity between groups. In our analysis of main effect that means activation in each condition, we found significant activation in right SMG, insula and middle frontal gyrus in healthy controls, but no significant activation in patients. In contrast, a comparison between groups did not show any difference of activation. In addition, there is a difference in analysis between local activity and connectivity. While the former is the degree of each local activity, the latter is the degree of their functional coordination. These are methodologically different and could lead to inconsistent results. In fact, some research studies showed different results of the analysis between local activity and connectivity (62, 63). In one study, using a reward learning task, significant activation in ventral striatum was found as a local activity in healthy controls compared to patients with schizophrenia. However, reduced functional connectivity was found between midbrain and insula in patients (62). In another research study using a reappraisal task, while less functional connectivity between prefrontal-amygdala was revealed in high psychosis-prone subjects, amygdala response to negative stimuli was decreased through reappraisal in rather low psychosis-prone subjects (63). These differences of the results between local activity and connectivity could represent the difference in analysis. Therefore, significant lower connectivity in patients cannot only be explained by the difference of the local activity.

One of the strengths of an fMRI study is that the brain network can be revealed beyond direct anatomical connectivity. The superior longitudinal fascicle III (SLF- III) originates from the SMG and terminates predominantly in the ventral premotor and prefrontal areas (64). Interestingly, the volume of the SLF-III is bigger in the right hemisphere than that in the left (65). As the prefrontal areas are closely connected with the basal ganglia, there is indirect anatomical connection between the SMG and the caudate, which reflects functional connectivity as shown in this study.

Our study has several limitations. First, this study involved an exploratory investigation using a small sample size and a low threshold (a cluster threshold which is lenient compared to FWE). In addition, we separately compared the connectivity and did not perform a model-based analysis to directly compare each condition. In the future, further research is required to precisely detect an aberrant agency network in schizophrenia patients. Second, our experimental design was a block design that was selected to weigh analysis of functional connectivity and is not appropriate for the analysis of each component of the agency task. Therefore, our analyses included many various components of the agency task, which may be one of the reasons why activation in the regions was shown only in the main effect. However, our previous study with the same agency task, but an event-related design, proved that the key component between pressing the button and perceiving SoA was associated with the insula and IPL by using direct comparison between the agency and color conditions (14). Third, our block design included many various components of the agency task resulting in a relatively low sensitivity of the main effect. Indeed, activation of caudate nucleus was not seen in our analysis of the main effect. However, as connectivity analysis revealed that the left caudate was activated with correlation to right SMG in the agency condition, we believe the failure to capture caudate activation in the analysis of the main effect was due to the low sensitivity of our block design. Fourth, it cannot be denied that abnormal functional connectivity may be secondarily associated with abnormal SMG in patients, however, there was no significant difference in the contrast analysis between the groups. Fifth, our findings might be influenced by medication. Hence, there is a trend-level correlation between contrast estimates and neuroleptic dosage. However, contrast estimates also had trend-level correlation with PANSS-P. As psychotic drugs were basically prescribed to treat positive symptoms, this relationship is complicated. In the future, further research is required to better investigate the relationship. Sixth, whether the abnormality is specific to schizophrenia is unclear because this study targeted only schizophrenia. In the future, comparisons among many various mental illnesses including major depressive disorder and bipolar disorder will be needed.

## Conclusion

In conclusion, the present study showed a deficit in the functional connectivity between the right SMG and the left CN in patients with schizophrenia. This dysconnectivity of the agency network may lead to self-disturbance through dysfunction in associative learning of SoA. The finding may provide a possible explanation for the reason abnormalities in the striatum cause schizophrenic symptoms including self-disturbance. In the future, a further research is required to detect the aberrant agency network more precisely in schizophrenia patients.

## Data Availability

All datasets generated for this study are included in the manuscript and/or the Supplementary Files.

## Ethics Statement

The procedure of this study was approved by the Institutional Review Board (IRB) of the Komagino Hospital. The study was carried out in accordance with the ethical guidelines set forth by the Declaration of Helsinki. We obtained a written informed consent from all of the subjects. Board certified psychiatrist (AK) who interviewed all participants ruled that they were capable of ethically and medically consenting for their participation in this research.

## Author Contributions

AK, TM, TO, YT, SU, and MK designed this study. AK, TO, YT, and TK conducted the statistical analyses. AK and TM wrote the first draft of the manuscript and edited the manuscript. SN, TY, MK, CN, YM, RD, TW, HK, and MM helped with data collection. All authors have approved the final manuscript.

### Conflict of Interest Statement

The authors declare that the research was conducted in the absence of any commercial or financial relationships that could be construed as a potential conflict of interest.
