# Supplementary material for: Dysconnectivity of the Agency Network in Schizophrenia: A Functional Magnetic Resonance Imaging Study
Source: Front Psychiatry. 2019 Apr 3;10:171. doi: 10.3389/fpsyt.2019.00171 (PMC6456683; doi:10.3389/fpsyt.2019.00171)
Supplement: Supplementary file 1 [file Table_1.DOCX]

Supplement

1.Main effect

**Agency condition.**

In healthy controls, analyses revealed increased activation of the right IPL (mainly supramarginal gyrus (SMG)), right insula, and right middle frontal gyrus as a main effect of the agency condition (p < 0.05, Family-Wise Error correction) (Figure 3). In patients with schizophrenia, the analyses revealed increased activation of the left supplementary motor area, left precentral gyrus, and left superior parietal lobule as a significant main effect of the agency condition.

**Color condition.**

In healthy controls, no significant main effect of the color condition was found. In patients with schizophrenia, the left precentral gyrus, left postcentral gyrus, left supplementary motor area, right inferior frontal gyrus, left insula cortex, right middle frontal gyrus, right postcentral gyrus, and left superior parietal lobule were found as a significant main effect.

| **Table. Regions showing a main effect of agency or color conditions in each group.** | | | | | | | |
| --- | --- | --- | --- | --- | --- | --- | --- |
|  |  |  |  |  |  |  |  |
| **Location** | | | **Coordinates (MNI)** | | | **z score** | **Voxels** |
|  |  |  | **x** | **y** | **z** |  |  |
| **Healthy Controls** | | |  |  |  |  |  |
|  | **Agency Main Effect** | |  |  |  |  |  |
|  |  | Rt. Inferior Parietal Lobule (Mainly BA40) | 48 | -39 | 44 | 5.13 | 41 |
|  |  |  | 45 | -49 | 42 | 5.00 |  |
|  |  |  | 41 | -56 | 54 | 5.02 | 5 |
|  |  | Rt. Middle Frontal Gyrus | 41 | 46 | -10 | 4.85 | 4 |
|  |  | Rt. Insula | 31 | 21 | 0 | 4.71 | 2 |
|  | **Color Main Effect** | |  |  |  |  |  |
|  |  | none | － | | | | |
|  |  |  |  |  |  |  |  |
| **Patients with Schizophrenia** | | |  |  |  |  |  |
|  | **Agency Main Effect** | |  |  |  |  |  |
|  |  | Lt. Supplementary Motor Area | -8 | 7 | 56 | 5.48 | 49 |
|  |  | Lt. Precentral Gyrus | -33 | -14 | 64 | 5.30 | 6 |
|  |  | Lt. Superior Parietal Lobule | -36 | -49 | 54 | 5.18 | 12 |
|  | **Color Main Effect** | |  |  |  |  |  |
|  |  | Lt. Precentral Gyrus | -36 | -14 | 64 | 5.95 | 107 |
|  |  |  | -33 | -11 | 54 | 5.28 |  |
|  |  | Lt. Postcentral Gyrus | -47 | -21 | 50 | 5.68 |  |
|  |  | Lt. Supplementary Motor Area | -5 | 0 | 54 | 5.28 | 100 |
|  |  |  | -8 | 0 | 62 | 5.11 |  |
|  |  | Rt. Inferior Frontal Gyrus | 48 | 7 | 30 | 5.16 | 10 |
|  |  | Lt. Insula | -29 | 21 | 0 | 4.98 | 4 |
|  |  | Rt. Middle Frontal Gyrus | 38 | 46 | 0 | 4.92 | 3 |
|  |  | Rt. Postcentral Gyrus | 45 | -28 | 56 | 4.80 | 6 |
|  |  | Lt. Superior Parietal Lobule | -29 | -56 | 52 | 4.78 | 6 |
|  |  |  |  | Extent threshold k=2 voxels | | | |

“2. Another connectivity analysis

As the Right Middle Frontal Gyrus was activated as the main effect of the agency condition, we also analyzed the data using BA46 (Middle Frontal Gyrus). However, there was no significant connectivity from this area.”
